# The Effects of Group Identity on Pro-environmental Behavioral Norms in China: Evidence From an Experiment

**DOI:** 10.3389/fpsyg.2022.865258

**Published:** 2022-03-25

**Authors:** Qinjuan Wan, Hongping Deng

**Affiliations:** ^1^School of Economics and Business Administration, Central China Normal University, Wuhan, China; ^2^Real Estate Economic Research Center of Hubei Province, Wuhan, China

**Keywords:** pro-environmental behavioral, social norms, group identity, experimental economics, simulations

## Abstract

This study experimentally evaluates the effects of group identity primed by property rights on pro-environmental behaviors (PEB) and social norms in an urban Chinese environment. The research in this paper expands the research perspective and method of domestic waste management and provides a theoretical basis for the establishment of a long-term mechanism of environmental treatment. We used two simple binary choice tasks that test the PEB and environmental types of individuals. This is one of the earliest tests for group identity and social norms in pro-environmental examinations in Chinese people. Our results reveal that (i) publicity and education have a significant positive effect on the development of individual and group pro-environmental behavioral norms; (ii) housing ownership has no differentiating effect on individual environmental behavior; and (iii) the development of social norms of pro-environmental behavior varies according to group conditions, which, in turn, determines individual environmental behavioral choices and types of environmental behavior. The results also suggest that PEB may be shaped and norms may be built by group conditions rather than group identity.

## Introduction

A new report shows that China released 27% of the global greenhouse gas emissions in 2020—larger than all developed countries combined.^1^ The environmental behaviors of Chinese people significantly affect China and even the worldwide environment. Environmental governance is not only associated with the mode of firm production but is also closely related to residents’ everyday lifestyles and environmental behaviors (Manisalidis et al., 2020). For example, residential life alone contributes 40% of total carbon emissions (Liu et al., 2011). Most previous studies have focused on the environmental protection behaviors of enterprises, but this paper focuses on the environmental behaviors of residents. The pro-environmental behaviors (PEB) of residents in different countries are different (Steg and Gifford, 2017), which is caused by many factors (national and ethnic cultural differences and the economic development level); hence, it is worthwhile to discuss this topic further (Mancha and Yoder, 2015; Morren and Grinstein, 2016).

Sharing attributes of public goods and eco-environmental resources have strong externalities in regard to their improvement, conservation, and maintenance. When a conflict arises between the environment and some small-scale individual economic interests, the externality of the individual resident’s environmental behaviors is reflected by a difficulty in spontaneously establishing environmentally friendly behavior norms among rational residents. This inevitably leads to the tragedy of the commons. Therefore, external intervention is needed. Although individuals’ everyday environmental behaviors have little to do with economic interests, intentional or unintentional non-PEB could combine to form an enormous total amount of carbon emissions, thereby increasing the damage to the environment.

After realizing the importance of individuals’ environmental behaviors, it is imperative to explore the causes of the weak motivation behind these behaviors before developing and advancing environmental behavior strategies. Non-economic measures can be effective in incentivizing residents to practice PEBs (van den Bergh, 2008; Hage et al., 2009). Humans are one of the most social species in nature (Henrich and Muthukrishna, 2021), and Durkheim (1895) believed that the activities of these “social beings” are subject to the constraints of common social norms. In contrast to mandatory law provisions, social norms refer to a set of commonly observed rules gradually established in people’s social practices and interactions; these rules are important manifestations of informal institutions (Cialdini et al., 1990). Group identity, which has been validated in other contexts, and its derivative group conditions may be a potential factor in influencing the cultivation of social norms (Lapinski and Rimal, 2005; Goldstein et al., 2008; Yin and Shi, 2021). Therefore, this paper innovatively introduces influencing factors such as group identity and group conditions into PEB research in China. Furthermore, the paper has practical significance for constructing a theoretical model.

Introducing domestic waste management rules and publicizing social norms on environmental protection are commonly used approaches to guiding individuals in establishing PEBs in practice, but their effectiveness for the residents’ group environmental behavior norms still needs to be quantitatively validated.^2^ For example, scholars influence publicity and education on PEB by using mediators such as attitudes (Mishal et al., 2017), values (Gilg et al., 2005), social atmosphere (Zhou et al., 2015), and awareness (Kirakozian, 2016). Meanwhile, other questions that need to be addressed also include the framing effect and group identity effect of the residents’ environmental behaviors. Input-based public resource provision and exploitation-based public resource consumption, which are common pool problems, are two different behavioral frameworks. Are there any differences between sorting and non-increasing environmental behavior due to varied frameworks? Additionally, does the difference in residents’ status primed by housing property ownership give rise to group identities of tenants or households and thus cause a difference in their environmental behaviors? To solve these problems, first, this study complements a laboratory experiment in which real community scenarios were simulated to inspire the social status identification of tenants and households. Second, we set group conditions and differentiated behavioral patterns to examine the residents’ environmental behaviors. Third, the micro-experimental data were collected and used to anticipate the internal logic of the residents’ environmental behaviors. Fourth, a computer simulation was conducted to explore the evolutionary path of the residents’ environmental behaviors. Finally, the “black box” of macro social behaviors was approached. This research is of great realistic significance for designing effective environmental governance mechanisms, cultivating the residents’ green lifestyle, and constructing a harmonious society.

This paper is organized as follows. Section “Literature Review” outlines the literature review. Section “Theoretical Hypotheses” proposes theoretical hypotheses. Section “Experimental Design and Procedures” describes the experimental design and procedures. Section “Results” presents the results. Section “Simulations” shows the simulation results. The last section offers the discussion and conclusion.

## Literature Review

This research includes two main aims: one is to investigate the relationship between environmental behaviors and publicity education, group identity, and group conditions, and the other is to distinguish the differences between waste sorting and reducing PEBs. Thus, the literature review of this article relates to the PEBs’ frameworks, publicity education, group conditions, and related research.

In this paragraph, we reviewed the social dilemma of PEB, comparing the different frameworks. Individual behaviors that benefit environmental protection are called PEBs. An individual’s environmental behaviors are positively correlated with the living environment but negatively correlated with a personal consumption utility. Homburg and Stolberg (2006) differentiate the individual’s PEBs into two categories: those maximizing positive effects (e.g., domestic waste sorting) and those minimizing negative effects (e.g., non-increasing the amount of waste). Practicing the PEBs of sorting and non-increasing domestic waste increases residents’ life costs and reduces their consumption usage. Czajkowski et al. (2014, 2017) suggested that the positive and negative causal relationship between individuals’ short-term interest conflict and environmental behaviors, as well as the low engagement of the individuals in PEBs and the social predicament of the public environment, is inevitable.

Here, we tried to return to the group identity research. Existing studies of group behaviors mainly adopt a paradigm that starts with realistic problems, draws conclusions from experimental data, and eventually achieves theoretical generalization. Group identity is a central concept in social psychology, sociology, anthology, and political science (McDermott, 2009). Group identity could be regarded as a factor that affects individual behavior, which is also a way to assess whether and to what extent people interact with in-group and out-group members (Chen and Li, 2009). The experimental research is highly effective in observing individual behaviors at a microscopic level and presents the interaction, aggregation, and trend toward a balance between group actions based on the characteristics of each group, making it an important instrument to decipher the “black box” for the emergence of macro social behaviors. Group identity originates from the individuals’ recognition of their statuses. In their studies of China’s social situations, Liu and Mao (2012) and Zhang and Yang (2017) pointed out that housing as the major component of urban household properties has already become an important indicator for wealth stratification, social differentiation, and interclass difference as a replacement for other traditional differentiation standards for social status. Through a laboratory experiment, Tajfel et al. (1971) validated that weak primary group identification will evoke biased group behaviors. Chen and Li (2009) and Saleem et al. (2018) found that, upon the completion of primary self-identification and weakness in-group identification, the increasing self-esteem, respect by others, empathy and value experience arising from out-group comparison, interaction, and other actions in line with group characteristics can further reinforce the sense of group identity, facilitate the stability and convergence of in-group behavioral patterns, and give rise to microscopic behavioral norms for both individuals and groups. Vazquez and Cortina (2018) defined the norms established by groups as “appropriate behavioral patterns,” which eventually form social norms.

Additionally, Ockenfels and Werner (2014); Krupka and Croson (2016), Abrams et al. (2021), and Wright (2021) found that the structural differences among group conditions in sets are also an important factor causing the differences in the direction and extent of group identification. Chen and Li (2009) manipulated the group status using painting preferences, and the players faced 3 kinds of potential cases, which were called the group conditions, no-group condition, in-group condition, and out-group condition.^3^ Based on existing research, Zhou et al. (2015) and Ke et al. (2018) examined the cooperation problem, finding that the more significant the in-group identity is, the higher the degree of division in the mixed group, which causes individuals to harm the interests of out-groups (even at the cost of their own interest), in addition to the repeated phenomenon of undermining the interests of the group and smaller cooperation inputs or larger consumption of non-co-operation. In combination with the social status differentiation model, the norm driver model, and the behavioral selection model, Akerlof and Kranton (2000) employed an economic game model to explain the behaviors of individuals themselves and others, as well as how identity and social norms drive the optimal solution to stable behaviors and the corresponding benefits. Li et al. (2019) revealed social status differentiation, a varied sense of responsibility, and group differentiation under a housing property. Based on a case study, Farrow et al. (2017) found significant effects of individuals’ selection of input and consumption environmental behaviors on the development of social norms. Lin and Xu (2014) and Wang and Sun (2019) also carried out an empirical study to reconfirm the far-reaching effects of housing property and social differentiation on the construction of the community environment.

To date, the existing literature on environmental behavior governance has largely focused on how the government, market, and communities cultivate PEB norms through co-governance. From the perspective of governmental regulation, Halvorsen (2012) explored different categories of policy and regulatory measures, such as establishing sophisticated environmental protection institutions and improving legislative and incentive mechanisms. Based on empirical analysis, Peng (2010) and Xu and Ling (2019) found that publicity and education of PEBs can cognitively improve an environmental protection utility, reinforce environmental protection drivers, and positively guide the practice of PEBs but may lead to non-robust and varied outcomes. From the perspectives of market prices, quotas, and mixed regulation mechanisms, Guo et al. (2017) and Wei et al. (2018) adopted an experimental method to discuss the effect of marketization on carbon emissions control and the social efficiency of product manufacturers and firms dealing with urban domestic waste in the tertiary sector. However, Ostrom (2009) confirmed that, while governmental and market regulation could address the vulnerability of environmental problems, community organizations could also play an important role in environmental governance. Ostrom et al. (2012) argued that community organizations are, undoubtedly, an important approach to compensate for the drawbacks of the former two factors. Under the concept of autonomous organization and governance, non-governmental organizations use organizational behavioral rules to establish collective behavioral norms and informal institutions to increase the initiatives of organizational members. Brzustewicz et al. (2021) explored multiple incentive mechanisms, finding that environmental values, beliefs, and reputation scores are effective monitoring mechanisms for safeguarding collective behavioral norms. In contrast to the heterogeneity in the effects of external constraints on group norms, such as under reward, incentive institutions, and marketization mechanisms, Allcott (2011) and Bolsen et al. (2014) found that household water and energy consumption behaviors across different groups show significant differences.

In summary, individual environmental behaviors among urban residents have drawn intensive scholarly attention. However, there are still some problems that need further consideration: First, the importance of giving equal emphasis to individual environmental behaviors and further highlighting firm environmental responsibilities. Second, the practical significance of the group stratification indicator of a housing property for researching the difference in PEBs—as well as the crucial role of group theory in interpreting residents’ environmental behaviors—has been underestimated. Third, the advantages of the experimental economic methodology in exploring microscopic behaviors have been overlooked. In this study, a low-cost laboratory experimental method was utilized, and participants were assigned the status of house tenants or purchasers to trace the dynamic vertical behavioral data and substantially reflect the dynamic, diachronic changes in the interaction, aggregation, and a trend toward the balance of the micro-individual and the group environmental behaviors. As such, the study aims to validate the mechanism of how the environmental behavioral framework, publicity education, and group identity influence the cultivation of environmental behavioral norms—with a view of benefiting the establishment in terms of long-term environmental governance mechanisms and steady policy advancements.

## Theoretical Hypotheses

### Is There a Framing Effect of Environmental Behavior Selection Under Spontaneous Conditions?

Public environmental resources bear attributes of natural public goods, such as “non-excludability” and “non-rivalry.” As individuals only obtain an insignificant fraction of resources in a large environment, residents tend to adopt a free rider-dominant strategy—such as non-PEB, promoting rational individual residents and groups to voluntarily put in public environmental resources, and exercise active abstinence measures. Since it is extremely difficult to fulfill common environmental welfare, the “tragedy of the commons” and “a prisoner’s dilemma” can frequently occur. Additionally, when confronted with different frameworks of environmental behavior selection, such as homogeneous waste sorting and non-increasing, people can exhibit distinct favoritism in their behavioral decision-making. Therefore, Hypothesis 1 (H1) is proposed.

H1: It is very difficult to establish spontaneous PEB norms, and spontaneous environmental behaviors vary across different environmental behavior frameworks.
(a). It is difficult to expect people to spontaneously establish PEB norms.
(b). People’s domestic waste sorting behavior is better than non-increasing behavior.

### How Does Publicizing Pro-environmental Behaviors Norms Regulate the Effect of Environmental Behavior Frameworks?

Publicity education of PEB norms can strengthen awareness of environmental protection, improve people’s environmental attitudes, and help them establish a correct sense of honor or disgrace toward environmental behaviors, as well as affect their willingness to engage in eco-environmental protection and endogenous behavioral drivers (Lu et al., 2020; Wang et al., 2020). By guiding residents’ PEBs and cultivating individual environmental protection awareness at the cognitive and subjective levels, publicity aiming to produce PEB norms represents an important approach to fulfilling these conditions. However, further exploration is still needed to determine to what extent the publicity of PEB norms facilitates sorting and non-increasing and whether publicity education causes differentiation or convergence in environmental behavioral frameworks. Therefore, Hypothesis 2 (H2) is proposed.

H2: Publicity education of environmental social norms can effectively direct people’s PEBs, and their effects vary across different behavioral frameworks.
a. Publicity education of environmental social norms can neither fundamentally change people’s environmental behaviors nor help establish complete PEB social norms.
b. Environmental social norms may have different effects on sorting and non-increasing.

### How do the Publicity Education of Social Pro-environmental Behaviors Norms and Group Identity Cause Interactive Effects on Environmental Behaviors Under Different Behavioral Frameworks?

Group identity theory interprets individual and group non-economic behaviors through group recognition, group status, and group comparison. Generally, individuals tend to classify others into the same or different groups based on their own characteristics and, at the same time, highlight status differences based on such identity characteristics (Eckel et al., 2010). Presently, housing assets are a major wealth component for the majority of residents, and tenant or purchaser identity is an important indicator of social stratification (Wu and Ge, 2019). The difference in tenant and purchaser identity may be reflected in the supply and acquisition behaviors of public goods. Therefore, Hypothesis 3 (H3) is proposed.

H3: After accepting environmental social norms, PEBs exhibit differences across tenant and purchaser identities.
a. Domestic waste sorting behavior varies across tenant and purchaser identities.
b. Non-increasing behavior varies across tenant and purchaser identities.

### Do Individual Environmental Behaviors Vary Across Group Conditions?

Group theory mainly involves two aspects: in-group favoritism and out-group biases. On the one hand, in-group dominance is highlighted as in-group conditions actively provoke mutually beneficial preferences among members, thus prompting them to exhibit in-group favoritism-based PEB norms. On the other hand, derogation or even hostility is directed toward out-groups. People tend to impose harsher punishments against non-cooperative behaviors of out-group members. The deterrent of harsh punishments also further stimulates people to exhibit out-group bias-based PEB norms (Wang, 2019; Wang and Sun, 2019).

H4: After accepting the publicity of environmental social norms, group conditions may change people’s environmental behaviors.
a. In-group conditions may change people’s environmental behaviors.
b. Out-group conditions may change people’s environmental behaviors.

## Experimental Design and Procedures

### Experimental Design

The purpose of the experimental design of this study is to determine the influence of publicity education,^4^ tenant-household social identities, and group conditions on the cultivation of norms under different environmental behavior frameworks. Individuals’ activities of daily living are inseparable from those of municipal solid waste (MSW), such as trash or garbage (clothing, food scraps, and batteries) from homes, schools, hospitals, and businesses. The source reduction presented a friendly view of the environmental behaviors. Our experimental design is based on Offerman and Sonnemans’s (1998) design. There are two types of source reduction behavioral decisions about MSW: one is sorting with a giving framework, and the other is reducing with a taking framework, both of which are completed in different situations. This experiment did some differences from the following aspects: (1) the number of the participants was increased, and the threshold for public goods was adjusted from 0.5 to 0.67. (2) The participants were asked to complete five 10-fold repetitions of decision interactions. We used a between-subject design, and the participants were always paired with another person from the same treatment. Table 1 presents our experimental design. Overall, 96 subjects were recruited for the experiment, as shown in Table 1. All the participants were students at China Central Normal University, and they were randomly equally distributed among the four treatments.

**TABLE 1:** Experimental design.

Treatment No.	Treatments	Monetary incentives	Social identities	Group conditions	N
➀	No publicity education, No-group conditions	0.015x	−	−	24
➁	Publicity education, No-group conditions	0.015x	−	−	24
➂	Publicity education, In-group conditions	0.015x	Households Tenants	Pure households	12
				Pure tenants	12
➃	Publicity education, Out-group conditions	0.015x	Households Tenants	Tenants-households mixed	24

This experiment mainly adopts two methods of publicity education to strengthen environmental social norms. One is to watch the eco-friendly video of MSW sorting and reducing pro-environmental behavior together. Two is to read the manual of Wuhan MSW management. After the automatic study, they were asked to complete self-testing to equip all the participants with consistent knowledge of pro-environmental behavior. In addition, for the group condition design, the participants should complete a slider competitive game together; they obtained corresponding scores through their own efforts, and the scores can be converted into benefits. Those who scored below the average are tenants, and the participants whose scores were above the average are the households. The classifications of social identities create two different group conditions: one is the in-group condition where all interaction participants were tenants or households; the other one is the out-group condition, where half of the participants are tenants, and the other half are households.

For the MSW sorting decisions, the participants’ revenue contains three parts: the individual endowments (60 experiment tokens), the costs of the sorting choice (60 experiment tokens), and the final public revenue. The final public revenue depends on the number of participants who choose sorting, if and only if the numbers are no fewer than 4 (including self), the public revenue is 245 experiment tokens; otherwise, it is zero.

The individual MSW sorting revenue:

Sorting⁢revenue=60-sorting⁢cost+final⁢public⁢revenue.

For the MSW reducing decisions, the participants’ revenue also contains the following elements: the public endowments (245 experiment tokens), the benefits of non-reducing choice (60 experiment tokens), and the final public loss. The final public loss depends on the number of participants who choose reducing, if and only if the numbers are no fewer than 4 (including self), the public loss is 245 experiment tokens; otherwise, it is zero. The details are shown in Table 2.

**TABLE 2:** Municipal solid waste (MSW) sorting and reducing payoff table.

The total numbers of sorting in the 6-person interactions (excluding self)							
Sorting decision		0	1	2	3	4	5
	Sorting	0	0	0	245	245	245
	Non-sorting	60	60	60	60	305	305

**The total numbers of Non-reducing in the 6-person interactions (excluding self)**							
Reducing Decisions		0	1	2	3	4	5
	Non-reducing	305	305	60	60	60	60
	Reducing	245	245	245	0	0	0

The individual MSW reducing revenue:

Reducing⁢revenue=245+non-reducing⁢benefits-final⁢public⁢loss.

The individual total MSW revenue:

Total⁢revenue=sorting⁢revenue+reducing⁢revenue.

### Experimental Procedures

The experiment was conducted in lab 419 of Experimental Economics, Nanhu Complex Building, Central China Normal University. Before the experiments, the participants were informed that they were completely anonymous during the whole experiment, and their personal information and decision-making information would be kept strictly confidential. No personal information, such as the name or student ID, was recorded during the experiments. After the experiments were completed, an independent experimental team would transfer the money through Alipay, and the experimental revenue was the personal information for all the participants. The participants were not allowed to communicate during the experiment.

Each experiment consists of three parts, and there are differences in the experimental process of different experimental treatments, as shown in Table 3 for details.

**TABLE 3:** Experimental procedures for four experiments.

Treatment No.	Part 1	Part 2	Part 3			Questionnaire
			No-group	In-group	Out-group	
➀	×	×	√	−	−	√
➁	√	×	√	−	−	√
➂	√	√	−	√	−	√
➃	√	√	−	−	√	√

Each participant was only asked to take part in the experiment one time, and the experiment consisted of three parts, each of which might be different. As the participants arrived at the laboratory, each randomly drew an ID card and then sat on the computer corresponding to the number. After the participants read the instructions for the first and second parts of the experiment, the experimenter read the whole experiment instructions again in combination with the software instructions. After reading the instructions in Part 3, the participants were given a piece of paper and a pencil to calculate the benefits of the experiment and to complete a self-test. After all the participants confirmed the experimental instructions, the experiment officially began.

In Part 1 of the experiment, all the participants had to be independent learners and study for 10 min by the video of publicity education related to the pro-environmental behavior of MSW and publicity manual on the sorting and reducing of MSW in Wuhan, and six test questions were completed within 3 min. In Part 2, the participants who completed the slider task with higher accuracy than average were awarded the title of household, who obtained tenant status below the average. In Part 3, 6 people were randomly divided into groups in each round, and 6 participants in the group completed a series of environmental decision-making tasks. The in-group condition means that all 6 participants in the group are households or tenants; the out-group condition means that there are 3 households and 3 tenants among the 6 participants in the group, and the no-group condition means that the 6 participants in the group have no rent-purchase status. The order of the environmental behavior decisions of MSW sorting and reducing appeared randomly. When participants make decisions, the historical information of their own and other participants’ behavior choices and average returns will be displayed on the decision page. In addition, the participants in the experimental group were asked to rate their sense of belonging to their household group and to the tenant group at the beginning and end of the third part of the experiment.

At the end of the experiment, the participants were asked to complete a questionnaire. The contents included human statistics, experimental strategies, a grouping of social groups, and difficulty of slider tasks. The total duration of each experiment was no more than 90 min, and the average income of the experiment was 65.8 yuan.

## Results

### Demographic Results

The demographic personal information of the participants was collected through a questionnaire. The majority of economics is denoted as 1, other majors as 0; 1 for girls and 0 for boys; Bachelor’s is marked as 1, other education levels are marked as 0; age is the average age of all the participants in each treatment. The statistical results are shown in Table 4. The Gamma independent homo-distribution test showed that the four variables mentioned above did not show significant differences among all experimental sites, ensuring that they were not the cause of the differences in experimental results.

**TABLE 4:** Demographic information of the four treatments.

Treatment code	Major	Gender	Education level	Age
➀	50.0%	87.5%	83.3%	20
➁	58.3%	70.8%	87.5%	21
➂	62.5%	95.8%	66.7%	21
➃	58.3%	83.3%	91.7%	20
Gamma test	0.18	0.20	0.24	0.14

### Pro-environmental Behavior and Frame Effects

Table 5 illustrates the frequency of 24 participants in each experimental session, choosing two kinds of PEB in 50 stages of decision-making. In treatment ➀, the frequencies of sorting and reduction were 45.93 and 35.07%, respectively. The results of the binary test show that the probability of spontaneous formation of the two kinds of pro-environmental behavior norms is far less than 67% (*p* = 0.00) of the condition of social norm formation, which shows that it is difficult to spontaneously form pro-environmental behavior norms, and Hypothesis 1(a) is valid. The last row of Table 5 shows that there are significant differences between the two PEB in each experimental session (*p* = 0.00), and the sorting behaviors of each experimental group are significantly better than the reducing behaviors, which indicates that the two environmental behaviors have a significant framing effect, which is consistent with Hypothesis 1(b), and is consistent with the previous experimental results (Andreoni, 1995; Sell and Son, 1997; Dufwenberg et al., 2011; Ellingsen et al., 2012).

**TABLE 5:** Pro-environmental behaviors (PEBs) and framework effects.

	Treatment codes				Bi-test
	➀	➁	➂	➃	= 67%
Sorting (%)	45.93	55.23	75.71	57.55	*p* = 0.00
Reducing (%)	35.07	27.46	61.20	48.75	*p* = 0.00
Non-parametric single factor test	0.00	0.00	0.00	0.00	−

### Publicity Education and Pro-environmental Behavior

In order to test the influence of publicity education on pro-environmental behavior, this part compares experimental treatment ➀ and experimental treatment ➁ with non-parametric tests, and the results are shown in Table 6. We found that publicity education significantly promoted the sorting behavior of MSW (*p* = 0.00) but had a negative effect on the behavior of reducing (*p* = 0.00). **Hypothesis 2** was only partially verified. Publicity education only has a positive impact on personal PEB to a certain extent, which is mainly reflected in the obvious increase in the number of people who choose MSW sorting (Zhou et al., 2015; Mishal et al., 2017; Zhang et al., 2020). However, we cannot improve the behavior of MSW reduction, perhaps because the amount of MSW is closely related to the quality of life. Reducing means low consumption, and the recognition obtained by reducing consumption is not enough to make up for the cost of sacrificing consumption (Xu et al., 2017; Wu and Ge, 2019; Xu and Ling, 2019). In the period of continuous improvement of living standards, publicity education alone is not enough to reduce people’s consumption desire, and the formation of PEB norms has a long way to go.

**TABLE 6:** A PEB test among four different treatments.

	Treatment codes				Non-parametric test			
	➀	➁	➂	➃	➀ vs. ➁	➁ vs. ➂	➂ vs. ➃	➃ vs. ➄
Sorting (%)	45.93	55.23	75.71	57.55	0.00	0.00	0.25	0.00
Reducing (%)	35.07	27.46	61.20	48.75	0.00	0.00	0.00	0.00

### Group Identity and Pro-environmental Behaviors

#### Manipulation Checks

Group identity can be measured by four indicators of the sense of belongingness to groups, which include the sense of belongingness to own a property group before the decision-making of Part 3, the sense of belongingness to own a property group after the decision-making of Part 3, the sense of belongingness to the other property group before the decision-making, and the sense of belongingness to the other property group after the decision-making. Here, two 0–10 value rating questionnaires (0 means nothing at all, 10 means extremely strong) are used to obtain the assignment of each index. Table 7 reports the average values of the four indicators. The results show that participants’ sense of belonging to their own property group is significantly higher than that of belonging to the other property group before and after the decision-making. It is proven that the second part of the slider task can successfully stimulate group identity (*p* = 0.00). In addition, there is no difference between the sense of belonging to the own property group and the sense of belonging to the other property group before and after the decision-making, which shows that the group identity of the participants will not be weakened by the decision-making task.

**TABLE 7:** The sense of belongingness.

Treatment codes	Before Part3		After Part3	
	The sense of belongingness to own property group	The sense of belongingness to the property group	The sense of belongingness to own property group	The sense of belongingness to the property group
➂	6.04	4.42	5.63	3.79
➃	6.46	4.54	6.58	4.46

#### Tenant-Household Identities and Pro-environmental Behavior

Table 8 shows the non-parametric single-factor test results of two PEBs and tenant-household identity. After the introduction of publicity education, the sorting behavior of tenants is slightly better than that of households (*p* = 0.06), without considering the group conditions. However, the tenant’s behavior of reducing is significantly better than that of the households (*p* = 0.00). Hypothesis 3 is basically verified (Xu and Ling, 2019). In terms of in-group conditions, there is little difference in sorting choice between households and tenants (*p* = 0.07). However, when out-group conditions are considered, the sorting behavior of households is significantly better than that of tenants (*p* = 0.00). This confirms the viewpoint that the increase in domestic waste is related to the quality of life. Compared with tenant owners, it has more wealth advantages and more consumption, so it is more difficult for them to reduce domestic waste.

**TABLE 8:** Tenant-household identities and pro-environmental behavior.

Frequencies of PEB Tenant-household identities	In-group conditions			Out-group conditions			Total
	Tenant	Household	Non-parametric test	Tenant	Household	Non-parametric test	Non-parametric test
Sorting (%)	73.50	77.93	0.07	63.33	51.75	0.00	0.06
Reducing (%)	55.92	66.50	0.00	38.56	58.93	0.00	0.00

#### Group Conditions and Pro-environmental Behavior

The element of publicity education was introduced into treatments ➁, ➂, and ➃, and then all the participants in those three treatments had the same knowledge of environmental protection. Under this premise, this paper carried out tests of group conditions and PEB, and the results are shown in Table 6.

(1) In-group conditions and PEB. By comparing experimental treatment ➁ with treatment ➂ by the non-parametric single-factor method, it is found that, after the condition of publicity education, compared with the no-group condition, the in-group condition has a significant positive effect on the two PEBs (*p* = 0.00). The setting of internal group conditions means that, in a pure household/tenant community, individuals with the same identity are more likely to compare and imitate one another and form some kind of PEB norms with group characteristics. Therefore, the effect of in-group favoritism on the two kinds of PEB is obvious, and the conclusion is consistent with Hypothesis 4 (a) in this paper.
(2) Out-group conditions and pro-environmental behavior. By comparing experimental treatment ➁ with treatment ➃ by the non-parametric single-factor method, it is found that, under the condition of publicity education, compared with the condition without a group, the out-group condition also has a positive effect on pro-environmental behavior, especially on the behavior of reducing (*p* = 0.00), while the number of people who choose sorting has increased, but it is not significant (*p* = 0.25). This conclusion is not completely consistent with Hypothesis 4 (b). The setting of out-group conditions means that in tenant-household binary mixed communities, individuals of different identities live together, and there are obvious differences in wealth and consumption levels among them, so the response is obviously different in the behavior of reducing (Bao and Li, 2020).

In addition, this paper also compares experimental treatment ➂ with treatment ➃ by the non-parametric single factor method and finds that the positive effect of in-group conditions on pro-environmental behavior is more significant than that of out-group conditions (*p* = 0.00). Under mixed living conditions, the cohesion within the group is low, the requirements of individuals for others with different identities are more stringent, and the prejudice of out-groups makes it difficult to form unified PEB norms.

### Probit Regressions

In this part, we used a probit model to regress all experimental data and investigate the marginal effect of publicity education and group conditions. The two explained variables are dummy variables, namely, sorting of domestic waste and reducing. Explanatory variables include publicity education, the number of experimental periods, the ending period (whether it is the last period or not), the PEB in the previous period (sorting/reducing decision), the total number of PEB in the previous period, their own income in the previous period, the average income of other participants in the previous period, and the interactive variables among the above variables.

#### Publicity Education and Pro-environment Behavior Regression Analysis

Table 9 illustrates the regression result of the influence of publicity and education on PEB. Publicity education increases the probability of PEB of domestic waste sorting and reducing, with 9.2 and 26.7% (a 1% confidence level), respectively. The coefficient of pro-environmental behavior in the previous period (d) shows that the PEB of individuals is stable. If sorting and reducing occur in the previous period, the probability of making the same choice in this period will increase by 31.8 and 28.7% (a 1% confidence level), respectively. The coefficient of the total number of PEB in the group in the last period shows that the probability of sorting in the current period will increase by 10% (a 1% confidence level) for each additional person in the group in the last period. Every time one person in the last group chose reducing, the probability of reducing in the current period decreased by 6.3% (a 1% confidence level), indicating that residents are more inclined to have “free ride” under the framework of reducing. The coefficients of pro-environmental behavior (d) in the last period of variable X publicity education are 0.212 and −0.146, respectively, which shows that publicity education has a positive effect on the stability of sorting behavior, but it has a negative effect on reducing behavior. In addition, individuals do not show cyclic effects and game-ending effects on sorting and reducing PEB.

**TABLE 9:** Probit regression results of publicity education on two PEBs.

Explanatory variable	Explained variable: PEB decisions	
	Sorting	Reducing
	Model 1	Model 2
Publicity education (d)	0.092*** (0.002)	0.267*** (0.039)
Period	−0.000*** (0.000)	−0.001 (0.001)
Last period (d)	−0.022 (0.048)	−0.020*** (0.002)
PEB in the previous period (d)	0.318*** (0.001)	0.287*** (0.021)
The numbers of PEB in the previous period	0.099*** (0.001)	−0.063*** (0.003)
Own payoff in the last period	−0.001*** (0.000)	0.000 (0.000)
The average payoff of others in the last period	0.001*** (0.000)	−0.000 (0.000)
Publicity education X PEB in the previous period (d)	0.212*** (0.001)	−0.146*** (0.021)
Publicity education X The numbers of PEB in the previous period	−0.050*** (0.001)	−0.056*** (0.009)
Own payoff in the last period	0.004*** (0.000)	−0.002*** (0.000)
Publicity education X The average payoff of others in the last period	−0.005*** (0.000)	0.001** (0.000)
No publicity education	0.497	0.277
Obs.	2,137	2,140
Pseudo R^2^	0.186	0.146

Marginal effects:(d) The marginal effect of discrete change from 0 to 1 for dummy variables; the standard errors in brackets are: **, *** significant at the level of 5 and 1%, respectively.

The above regression further proves our experimental results: publicity education has a positive impact on PEB, but the impact on sorting is significantly higher than that on reducing. It can be seen that publicity education can reduce opportunism and “free riding” unfriendly environmental behaviors and cultivate PEB norms.

#### Group Conditions and Pro-environment Behavior Regression Analysis

Table 10 shows the regression results of the influence of group conditions on PEB.

**TABLE 10:** Probit regression results of group conditions on two PEBs.

Explanatory variable	Explained variable: PEB decisions	
	Sorting	Reducing
	Model 1	Model 2
In-group conditions (d)	0.476*** (0.001)	−0.429*** (0.036)
Out-group conditions (d)	0.005 (0.006)	
Periods	0.000 (0.000)	−0.001 (0.001)
Last period (d)	−0.052*** (0.011)	−0.055* (0.024)
PEB in the previous period (d)	0.495*** (0.006)	0.120*** (0.008)
The numbers of PEB in the previous period	0.043*** (0.000)	−0.141*** (0.006)
Own payoff in the last period	0.003*** (0.000)	−0.001*** (0.000)
The average payoff of others in the last period	−0.003*** (0.000)	0.001*** (0.000)
In-group Conditions X PEB in the previous period (d)	0.409*** (0.004)	0.421*** (0.036)
Out-group Conditions X PEB in the previous period (d)	−0.592*** (0.040)	0.170*** (0.023)
In-group Conditions X The numbers of PEB in the previous period	−0.143*** (0.002)	0.170*** (0.023)
Out-group Conditions X The numbers of PEB in the previous period	0.126*** (0.012)	−0.001* (0.003)
In-group Conditions X Own payoff in the last period	−0.001*** (0.000)	−0.001 (0.001)
Out-group Conditions X Own payoff in the last period	−0.013*** (0.001)	−0.002*** (0.000)
In-group Conditions X The average payoff of others in the last period	−0.000** (0.000)	0.001 (0.001)
Out-group Conditions X The average payoff of others in the last period	0.013*** (0.001)	0.00*** (0.000)
No-group conditions	0.660	0.449
Obs.	3,225	3,225
Pseudo R^2^	0.281	0.361

marginal effects:(d) The marginal effect of discrete change from 0 to 1 for dummy variables; the standard errors in brackets are: *, **, *** significant at the level of 10, 5, and 1%, respectively.

(1) Compared with the no-group conditions, the in-group conditions increase the probability of domestic waste sorting by 47.6% but decrease the probability of reducing it by 42.9%. However, the influence of out-group conditions on the two environmental behaviors can be neglected.
(2) Group conditions can alleviate the ending effect. Compared with the no-group conditions, the probability of choosing sorting and reducing at the end of the group decreased by 5.2 and 5.5%, respectively.
(3) The PEBs are stable under in/out-group conditions. The participants who chose sorting/reducing PEBs in the previous period increased their probability of continuing to choose them by 49.5 and 12%, respectively, in this period. The coefficients of the last environmental behavior (d) of the in-group Condition X in the sorting and reducing decision regressions are 0.409 and 0.421, respectively, indicating that the in-group conditions can promote the stability of PEB. The coefficients of the environmental behavior (d) in the last period of the interactive out-group condition in the regressions of sorting and reducing decision-making are −0.592 and 0.17, respectively, which indicates that the out-group condition will weaken the stability of sorting behavior, but it has a positive effect on the stability of reducing behavior. The participants who chose two PEBs in the previous period increased their probability of continuing to choose them by 49.5 and 12%, respectively, in this period. The coefficients of the last environmental behavior (d) of the in-group condition in the sorting and reducing decision regression are 0.409 and 0.421, respectively, indicating that the intragroup condition can promote the stability of pro-environmental behavior. The coefficients of the environmental behavior (d) in the last period of the interactive external group condition in the regression of sorting and reducing decision-making are −0.592 and 0.17, respectively, which indicates that the out-group condition will weaken the stability of sorting behavior, but it has a positive effect on the stability of reducing behavior.
(4) Participants’ PEB decisions will be influenced by the total number of PEBs and group conditions in the previous group. The correlation coefficients between the two PEBs of the participants in the current period and the total number of PEB in the previous group (0.043, −0.141) show that individuals are more willing to follow the positive behaviors of most people in sorting. However, they prefer to be free riders when they are in a reducing framework. The comprehensive coefficients of the in-group conditions and the total number of PEBs in the last period in the sorting and reducing regressions were 0.376 (0.476 + 0.043 − 0.143) and −0.4 (0.429 − 0.141 + 0.170), respectively. This result indicated that their influence on the current sorting decision was positive, but their influence on the reducing decision was negative. The comprehensive coefficients of the out-group conditions and the total number of environmental behaviors in the previous period in the sorting and reducing regressions were 0.169 (0.043 + 0.126) and −0.142 (−0.141 − 0.001), respectively, indicating that their influence on the current sorting decision-making was positive, but their influence on the reducing decision-making was negative.

In other words, the results of the regressions prove the stability of the experimental results. That is, the sorting probability of personal domestic waste under the in-group conditions is significantly higher than that of other cases, and it is more stable. Participants adopt reciprocity strategies to sort to improve publicity resources. Group identity stimulation is a favorable factor to promote personal PEBs. However, different group conditions have different paths to stimulate individual PEBs. In addition, the influence of group conditions is also different under different environmental behavior frameworks.

## Simulations

The results of experiments and regression show that publicity education and group conditions are both important factors in adjusting individual PEBs and contribute to the formation of PEB norms, while effective social norms can straighten out the logic of collective actions and promote public environmental welfare. In a given situation, the formation of personal environmental behavior norms is determined by factors such as their own historical environmental behavior, the historical environmental behavior of others in the group, their own income changes, and the average income changes of others. Next, we used computer simulation to explore the evolution path of publicity education and environmental behavior decision-making under corresponding group conditions.

### Pro-environmental Behavior Logic of the Individual Level

Before the implementation of computer simulation, this paper determines the decision logic of individual environmental behaviors based on experimental data. Because of the difference aversion, the combination situation of individual income changes is the decisive factor of environmental behavior. Therefore, in this paper, two kinds of environmental behaviors of domestic waste sorting and reducing are regarded as explained variables. Publicity education, group conditions, ending period, the combination of the income difference between the previous two periods (△π_*i*_, △π_−*i*_) and the interactions between them are explanatory variables. Here, if △π_*i*_ is greater than zero, noted as “added,” and if △π_*i*_ is not greater than zero, noted as “no added,” and so on, for each of the △π_−*i*_. The most intuitive probit regression models are used to deduce the participants’ environmental behavior logic, and the regression results are shown in Tables 11, 12.

**TABLE 11:** Probit regressions of publicity education and the combination of income changes on two PEBs.

Explanatory variable	Sorting	Reducing
	
	(1)	(2)
Publicity education (d)	0.049*** (0.001)	−0.110*** (0.000)
(no added, added) (d)	0.000 (0.002)	−0.213*** (0.000)
(added, no added) (d)	0.059*** (0.001)	−0.021*** (0.000)
(added, added) (d)	0.225*** (0.000)	−0.041*** (0.000)
Publicity education X (no added, added) (d)	0.221*** (0.001)	0.112*** (0.000)
Publicity education X (added, no added) (d)	0.077*** (0.001)	−0.205*** (0.000)
Publicity education X (added, added) (d)	−0.026*** (0.001)	0.118*** (0.000)
Last period (d)	−0.057 (0.030)	−0.078***(0.009)
No Publicity education X (no added, no added)	48.6%	28.8%
Obs.	1,914	1,915
Pseudo R^2^	0.0369	0.0393

Marginal effects:(d) The marginal effect of discrete change from 0 to 1 for dummy variables; the standard errors in brackets are: *** significant at the level of 1%.

**TABLE 12:** Group condition and the combination of income changes Probit regressions on two PEBs.

Explanatory variable	Sorting	Reducing
	
	(1)	(2)
In-group condition (d)	0.344*** (0.001)	0.521*** (0.001)
Out-group condition (d)	0.153*** (0.001)	0.305*** (0.001)
(no added, added) (d)	0.197*** (0.002)	−0.141*** (0.000)
(added, no added) (d)	0.122*** (0.001)	−0.290*** (0.001)
(added, added) (d)	0.185*** (0.001)	0.080*** (0.001)
In-group Condition X (no added, added) (d)	−0.170*** (0.001)	−0.451*** (0.000)
In-group Condition X (added, no added) (d)	−0.545*** (0.001)	−0.172*** (0.005)
In-group Condition X (added,added) (d)	−0.282*** (0.003)	−0.339*** (0.002)
Out-group Condition X (no added, added) (d)	−0.169*** (0.003)	−0.242*** (0.001)
Out-group Condition X (added,no added) (d)	−0.419*** (0.002)	−0.238*** (0.004)
Out-group Condition X (added,added) (d)	−0.201*** (0.000)	−0.165*** (0.002)
Last period (d)	−0.050(0.026)	−0.078***(0.026)
No-group Condition X (no added, no added)	63.6%	−
Obs.	2,876	2,876
Pseudo R^2^	0.065	0.187

Marginal effects:(d) The marginal effect of discrete change from 0 to 1 for dummy variables; the standard errors in brackets are: *** significant at the level of 1%.

Table 11 shows the logic of individual environmental behavior decision-making under publicity education. With the deepening of publicity education, the practice of domestic waste sorting has an obvious increasing trend, but the behavior of reducing has not improved significantly. The coefficients of (added, no added) and (added, added) of 0.059 and 0.225 indicate that, without considering the average changes of other people’s income in the previous two periods in the group, there is a positive relationship between their own income changes in the previous two periods and the current sorting decision. The coefficient of (no added, no added) is −0.213, which indicates that the changes of self-income in the previous two periods are negative, and the average changes of other people’s income in the previous two periods are positive, which can significantly reduce the probability of individual environmental behavior decision-making of reducing. The coefficient of the interaction of (no added, added) (d) and publicity education is 0.112, which shows that publicity education can alleviate the negative effect of income change comparison.

Table 12 shows the logic of individual environmental behavior decision-making under group conditions. Both in- and out-group conditions can significantly improve the probability of sorting and reducing domestic waste. The cases of (no added, no added), (added, no added), and (added, added) all have a positive influence on sorting decision-making, but the influence on decision-making reduction is not significant. The coefficients of the interactive variables of the combination of group conditions and income changes are all negative, which indicates that there is a mutual weakening relationship between these two variables.

### Simulation Results

There are 16 (2×2×4) situations among publicity education, group conditions, the combination of income changes, and the probability distribution of two PEBs in each situation, which can be regarded as the logic of environmental behaviors in a specific situation, as shown in Table 13.

**TABLE 13:** Probability distribution of PEBs under income changes, publicity education, and group conditions.

	Publicity education				Group condition			
	NO Publicity education No group condition		Publicity education No group condition		Publicity education In-group condition		Publicity education Out-group condition	
	Sorting	Reducing	Sorting	Reducing	Sorting	Reducing	Sorting	Reducing
(No added, no added)	49%	29%	54%	18%	97%	96%	78%	75%
(No added, added)	49%	8%	76%	8%	100%	30%	82%	37%
(Added, no added)	55%	27%	68%	15%	55%	15%	48%	22%
(Added, added)	72%	25%	70%	26%	87%	52%	76%	67%

**FIGURE 1:**
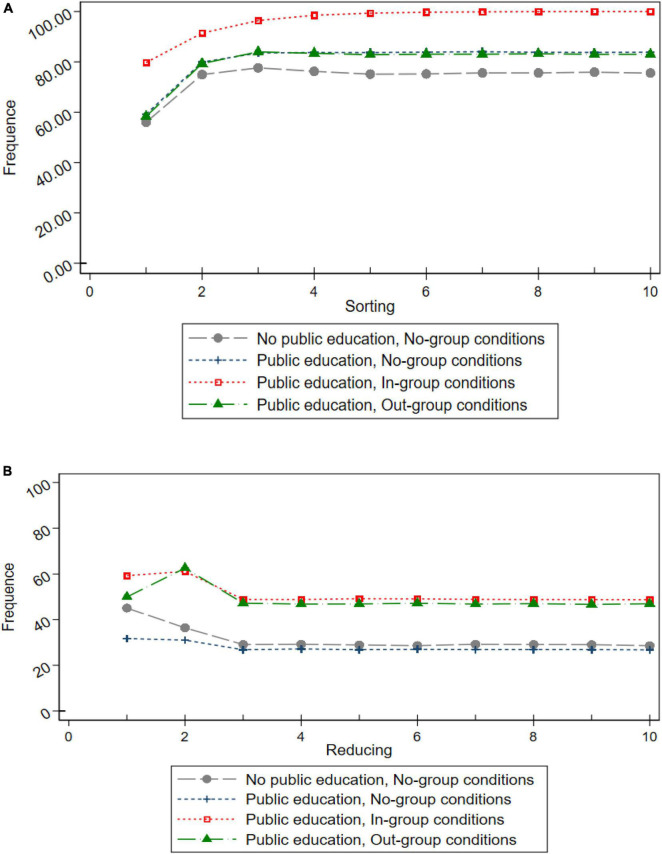
The trend of the probability distribution of pro-environmental behaviors (PEBs). **(A)** sorting (simulations). **(B)** Reducing (simulations).

The computer simulation process is as follows: (1) extracting data randomly. Taking the experimental data as the parent data, 10,000 random retrievable extractions were made for each experimental treatment by the Stata bootstrap method. Each extraction included the data of five 10-subgames completed by six participants, and only the environmental behavior decision data of the first two periods of each participant were kept. (2) Simulating the environmental behavior decisions from the third to tenth periods. The participants’ third-stage environmental behavior decision-making is determined by the situation under the combination of the characteristics of the experimental treatment and the combinations of income changes in the previous two periods and is determined by the probability distribution of environmental behavior under this situation. By analogy, the final simulation produced 1.2 million (6×10×5×10,000×4) (= 6 * 10 * 5 * 10,000 * 4) observations. (3) Deriving the evolutionary path of individual pro-environmental behavior. The evolution path is shown by the probability distribution trend of pro-environmental behavior, and the results are shown in Figure 1.

## Conclusion, Suggestions, and Prospects

The World Bank report predicts that with rapid urbanization, population growth, and economic development, the global waste volume will reach 2.59 billion tons in 2030 and 3.4 billion tons in 2050. Currently, the top three countries producing municipal solid waste (MSW) are the United States of America (258 million metric tons), China (220 million metric tons), and India (169 million metric tons). Five of the top 10 domestic waste producers are developing countries (China, India, Brazil, Indonesia, and Mexico) (Statista, 2020). Different geographical cultures, living habits, levels of consumption, and economic development are the roots of differences in waste generation and composition. Developed countries often produce much more MSW *per capita* than developing countries and third world countries because the waste generation rate depends on the economic and social prosperity of a country, such as the United States, 2.58 kg/capital/day. In other words, the *per capita* generation of MSW varies among different income groups across the world. On the other hand, the proportion of inorganic components in MSW increases with the increase in gross national income (Baker, 2012; Aleluia and Ferrão, 2016). Organics can account for 65% of MSW from low-income groups, compared with only a quarter from high-income groups (Baker, 2012). Furthermore, the composition determines the heating value of MSW. The heating value of MSW in developing countries is low, mainly because of the high content of organic matter and water in MSW (Gerassimidou et al., 2013; Kumar and Samadder, 2017).

Internationally, the levels of MSW management are the same, that is, reducing/reducing sources, reuse, recycling/composting, waste recycling/energy, and disposal/landfill. However, the present status of MSW management methods varies from country to country, and the efficiency of MSW management depends on the characteristics, composition, and heterogeneity of wastes. Generally, it can be inferred that the recovery rate of MSW in developed countries is relatively high, which has a positive impact on reducing the generation of MSW. By region, North America, Europe, and Central Asia have high recycling proportions (e.g., Germany and Korea have 62 and 61% waste recycling, respectively). Burning rates are high in East Asia and the Pacific (Japan is one of the leading countries for waste incineration). South Asia is the worst region for open dumping, with a larger proportion than sub-Saharan Africa (Bangladesh and Thailand); and compost disposal is high in South Asia and Europe and Central Asia (the United States and China have higher landfilling percentages of 53.8 and 60.16%, respectively) (Mian et al., 2017). For each country, no matter what kind of MSW management method is chosen, source reduction is the key first step, and MSW classification and MSW emission reduction are the main approaches. Therefore, it is of academic value and practical significance to study how to encourage people to participate in MSW classification and reduction.

As the report pointed out, many cities in China are facing a serious crisis of garbage siege (more than two-thirds).^5^ Incineration is one of the major ways to dispose of MSW, and air pollutants and greenhouse gases emitted from incineration are two main problems that may cause severe harm to human health (Williams, 2005; Yang et al., 2012). In addition, it is notable to reduce greenhouse gas emissions to achieve a promise for carbon peaking and neutrality. The technology of MSW incineration still has a long way to go in China. This fact, once again, reinforces the importance of source reduction, which includes MSW sorting and reduction. Classifying solid waste before disposal will reduce greenhouse gas emissions by 24%. This article focused on the pro-environmental behavioral norms (MSW sorting and reducing) cultivation of residents in cities, and topics, such as publicity education, group identity, and group conditions, are covered in more detail.

Based on an experiment on the environmental behaviors of subjects, this paper validates the predicaments and framing effect of spontaneous environmental behaviors and the effects of publicity and education and group conditions on individual environmental behaviors. Regression and computer-based simulations were conducted to test the robustness of the results. The following conclusions were drawn:

(1) The experimental results show that, without intervention, it is very difficult for individuals and groups to develop PEBs and norms. Despite a high correlation between the two domestic waste environmental behaviors, the framing effect was still highly distinct, and the sorting behavior was significantly better than the non-increasing behavior, with the former being more stabilized than the latter in in-group cooperation. In addition, the tenant or purchaser identity further differentiated the two environmental behaviors, with property owners being more unwilling to reduce their consumption levels to lower the output of domestic waste.
(2) After introducing the publicity and education variable, it was found that publicity and education could improve individual and group environmental behaviors to a certain extent but with different effects. Publicity and education helped improve the probability of the individuals’ sorting PEB, but with a limited effect on the non-increasing behavior. From the long-term evolution results, publicity and education could not effectively increase the probability of non-increasing behavior.
(3) After the groups were classified by tenant and purchaser identities, the group conditions improved individual and group environmental behaviors. Environmental behaviors exhibited significant in-group favoritism, and in-group conditions had a facilitating effect on the stability of both environmental behaviors. However, as seen from the evolution results, the non-increasing behavior has been flat. No distinct out-group bias was found in the out-groups. The out-group conditions only had a facilitating effect on the stability of the non-increasing behavior, and the long-term evolution results confirmed the experimental conclusion. This means that long-term interaction creates a probability of punishing non-cooperative behaviors and that the potentially high deterrent of out-group conditions increases the probability of the occurrence of PEBs more than otherwise.

Based on the empirical discussion and experimental conclusions stated above, the following suggestions are proposed: ➀ the construction of zero-waste cities requires the formation of PEB norms among urban residents, but PEB is difficult to develop spontaneously, and the framing effect also plays a role. While introducing MSW management rules, it is also necessary to increase governance efforts and exercise separate governance over sorting and non-increasing behaviors. As domestic waste is largely produced within communities, further attention should be given to different environmental behaviors caused by tenant and purchaser identities in communities. ➁ Publicity and education are effective approaches to cultivate residents’ PEB norms and are characterized by good economic efficiency and high feasibility. Given the positive effects of publicity and education on waste sorting behavior, governmental departments may step up their efforts and expand the scope of such publicity. However, non-increasing behavior entails individuals’ quality of life; thus, the effect of publicity and education is limited. Introducing market approaches can be considered to impose constraints on affluent groups, such as using the quota system to fulfill higher payments for higher emissions. ➂ The structures of urban community popularity are diversified due to tenant and purchaser identities. Thus, measures, such as economic incentives, social engagement, and strict law enforcement, should be adopted based on the characteristics of the residents’ environmental behaviors in mixed or non-mixed communities to normalize their environmental behaviors and ensure the fulfillment of collective environmental protection actions. ➃ The positive social norms that are not closely related to economic factors are easy to form (MSW classification), but the positive social norms that are highly related to economic factors are more difficult to form, which is similar to the PEB norms of reducing (those results are consistent with the World Bank report, which implies high-income people would be less likely to practice the PEB norms of reduction). Therefore, this study can apply a series of topics about the effects of wealth on the formation of social norms, which are constructed on social dilemmas and cooperation issues.

Compared with previous related studies, the innovation of this study and its significance to follow-up research is mainly reflected in three aspects: first, in our study of pro-environmental behavior norms, we pay much attention to behavioral economic theories and methodologies, which combine the two PEB of source reduction with the two frameworks of public goods. It provides a research paradigm and enriches the literature on pro-environmental behavior. Second, a large number of studies on pro-environmental behavior issues focus on static behavior in a certain situation, while this study places more emphasis on the dynamic process of pro-environmental behavior adjustments. Clearly, our study places a higher value on quantitative research rather than qualitative research. This study could provide a reference for future pro-environmental behavior research with respect to the analysis. Third, the issues of environmental behavior research are relatively simple, with only one kind of behavior. However, our study asks participants to make two PEB at once, which implies that the two decisions are not completely independent. This design in our experiments gives more chances to perform comparative analysis from many angles. This study might bring some fresh ideas to create experiments of PEB.

The limitations of our study and suggestions for subsequent research can be summarized as follows: In this paper, a laboratory experimental method was used to examine the factors influencing individual environmental behaviors. The conclusions of this research are internally valid. Future research may focus on field experiments on property owners and tenants in real communities instead of undergraduate students. In addition, this paper mainly focuses on the effects of domestic waste management rules and publicity and education on urban residents’ environmental behaviors. However, other economic approaches, such as quota allocation and pricing, can also exert important effects on individual environmental behaviors. Future studies may consider incorporating market regulation approaches into the design mechanism for cultivating individual environmental behavior norms.

## Data Availability Statement

The raw data supporting the conclusions of this article will be made available by the authors, without undue reservation.

## Ethics Statement

The studies involving human participants were reviewed and approved by the Central China Normal University. Written informed consent for participation was not required for this study in accordance with the national legislation and the institutional requirements.

## Author Contributions

QW and HD contributed to conception and design of the study. QW collected the data, performed the statistical analysis, and wrote the first draft of the manuscript. Both authors contributed to manuscript revision, read, and approved the submitted version.

## Conflict of Interest

The authors declare that the research was conducted in the absence of any commercial or financial relationships that could be construed as a potential conflict of interest.

## Publisher’s Note

All claims expressed in this article are solely those of the authors and do not necessarily represent those of their affiliated organizations, or those of the publisher, the editors and the reviewers. Any product that may be evaluated in this article, or claim that may be made by its manufacturer, is not guaranteed or endorsed by the publisher.

## Footnotes

1 Data source link: http://english.mee.gov.cn/accessed November 10, 2021.

2 In 2000, China piloted domestic waste sorting in eight cities, including Beijing, Shanghai, Guangzhou, and Shenzhen, by education and advocacy, putting assorted waste bins on the streets as well, with poor results. In 2017, the National Development and Reform Commission and Ministry of Housing and Urban-Rural Development issued a plan for garbage sorting, urging 46 cities to set up a basic system of laws and regulations on waste sorting by the end of 2020. (Source: http://www.gov.cn/zhengce/content/2017-03/30/content_5182124.htm).

3 No-group condition: subjects had no group status information on their counterpart. In-group condition: subjects informed that their counterpart had the same group status as them. Out-group condition: subjects informed that their counterpart’s group status was not like them.

4 The publicity education means the norms, which convey that waste sorting and reducing are pro-environment, called “injuctive norms” by psychologists (Cialdini et al., 1990).

5 Data source: https://www.hbzhan.com/news/detail/107410.html.
